# Value creation and the internal goods of business

**DOI:** 10.3389/fsoc.2022.980816

**Published:** 2023-01-05

**Authors:** Caleb Bernacchio, Robert Couch

**Affiliations:** ^1^College of Business, California State University, Monterey Bay, Seaside, CA, United States; ^2^Business Department, Earlham College, Richmond, IN, United States

**Keywords:** business, MacIntyre, Alasdair, strategic management, mutual benefit

## Abstract

In his early work, Moore argues that business itself was a MacIntyrean practice. He later rejected this view in response to criticisms from Beadle and others. Most subsequent work, including that of Moore, adopted a view of organizations, including firms, as institutions that house a core practice. We first recount Moore's early view, defend and it from various criticisms. We then briefly review research in management and finance arguing that this research supports a view of business consonant with Moore's early view. Thus, we argue that business is a distinct practice that integrates various productive and auxiliary practices to facilitate mutually beneficial transactions. We conclude by discussing implications of this view, noting that it might be viewed as a classical liberal appropriation of the MacIntyrean framework, and arguing that it poses a challenge to MacIntyreans working with a neo-Aristotelian perspective.

In applying virtue ethics to business contexts, MacIntyre's (2007) account of social practices has been very influential (Moore, 2017). This influence can largely be attributed to the critical nature of his account, which captures the moral precarity associated with an exclusive focus on obtaining external goods, such as money, status, and power. However, this critical aspect also implies a negative view of business, which MacIntyre does not consider to be a practice. In order to apply MacIntyre's framework to business ethics in a constructive way, many researchers have followed Moore (2005) in arguing that while business itself is not a practice, businesses (i.e., particular firms) are often the bearers of practices (Moore, 2005, 2017; Beadle and Moore, 2006; Moore and Beadle, 2006; Tsoukas, 2018). While this approach has proven to be a fruitful in business ethics, and in some other strands of management research, we maintain that it does not sufficiently take account of the inherent normative element in business, that is, its ethical core, treating it, instead, as amoral at best (see Wicks, 1996). Likewise, a focus on business as itself a practice promises to provide new avenues for integrating research in strategy and finance with business ethics (see Bernacchio et al., 2022).

We argue that business is, indeed, a practice whose internal goods are realized by facilitating mutually beneficial exchange between businesses and customers (Bruni and Sugden, 2013). Our account of business is closely aligned with recent trends in the strategic management literature suggesting that business is a distinct activity that integrates heterogeneous resources, forms of knowledge, and practices in the service of value creation and value capture (Grant, 1996; Tsoukas, 1996; Teece, 2007; Foss and Klein, 2012). Moreover, in order to create value for customers, and to survive the pressures of competition, businesses must promote collaboration and innovation, activities which are supported by exercising the virtues, especially the virtues of creativity, cooperation, and sustained focus. Our account of business as a practice fits within the neo-Aristotelian tradition, insofar as we as we adopt MacIntyre's conception of social practices. However, our focus on mutual benefit as the underlying aim of business draws on the tradition of classical liberalism, going back to Smith (2003) and developed more recently by a number of scholars (McCloskey, 2007; Tomasi, 2012; Bruni and Sugden, 2013; Otteson, 2019). Although invoking the liberal concept of mutual benefit makes our account of business more vulnerable to criticism from a neo-Aristotelian perspective, we maintain that on both empirical and normative grounds, it represents a worthwhile improvement.

To support and elaborate our account of business as a practice, we also discuss the contribution of finance to business. Whereas MacIntyre has focused particular criticism on finance (MacIntyre, 2015), we suggest that finance plays—or, can play—a crucial role in supporting business as a practice, by facilitating collaboration and innovation through the integration of knowledge and resources across disparate actors and domains. The contribution of finance to business is particularly evident in the collaboration that occurs between venture capitalists and entrepreneurs, and between dedicated investors and corporations which host multiple core practices. We do not deny the many negative examples and problems in finance and business, but we propose that these problems are largely the result of the practice of business being corrupted by an exclusive focus on value capture and rent-seeking, detached from the aim of value creation, where the virtues of business are exercised.

## Customer value and the internal goods of business

In *After Virtue*, it is clear that MacIntyre intends to contrast his account of practices, narratives, and tradition with the social context of modernity, including that of bureaucratic corporations. Specifically about practices, MacIntyre (2007, p. 227) says:

As, and to the extent that, work moves outside the household and is put to the service of impersonal capital, the realm of work tends to become separated from everything but the service of biological survival and the reproduction of the labor force, on the one hand, and that of institutionalized acquisitiveness, on the other. [...] The means-end relationships embodied for the most part in such work—on a production line, for example—are necessarily external to the goods which those who work seek; such work too has consequently been expelled from the realm of practices with goods internal to themselves.

Thus, there is little question as to MacIntyre's answer to the question of whether business is a practice. As he says even more succinctly, “Pleonexia, a vice in the Aristotelian scheme, is now the driving force of modern productive work” (MacIntyre, 2007, p. 227). Likewise, MacIntyre (2007, p. 86) says there are two aspects to managerial expertise, “the aspiration to value neutrality and the claim to manipulative power.” As such, management, the central role in any business with at least a handful of employees, is defined in purely instrumental terms, as lacking any constitutive ethical commitments. MacIntyre's view, at least as presented within *After Virtue*, is that business within a capitalist context tends to be a purely instrumental activity, where productive work is alienating and management tends to be a matter of manipulating employees in order to achieve financial objectives. As a result, MacIntyre (2007, p. 227) says, in modernity “practices have in turn been removed to the margins of social and cultural life.”

Despite MacIntyre's clear opposition, and (Moore, 2005, 2017) later shift to a weaker thesis (*viz*., that businesses are practice-institution combinations), Moore (2002) argues in an early paper that business is in fact a MacIntyrean practice. MacIntyre (2007, p. 187) defines a practice as “any coherent and complex form of socially established cooperative human activity through which goods internal to that form of activity are realized in the course of trying to achieve those standards of excellence which are appropriate to, and partially definitive of, that form of activity, with the result that human powers to achieve excellence, and human conceptions of the ends and goods involved, are systematically extended.” Using the example of a retail business, Moore (2002, p. 24) says:

Retailing involves all of the usual functions of business—purchasing stock, employing staff, purchasing or renting premises, out-of-store advertising, displaying and selling goods, tracking stock with computer systems, recording sales and feeding the information into the accounts, accounting, financial control, and so on. Retailing involves the integration of all of these elements into a holistic activity.

Here, a core idea emerges concerning the nature of business as a practice: it involves the ongoing integration of different business functions. Thus, Moore (2002, p. 24) concludes, when business is viewed in this light as a “holistic activity [...] it accords fully with MacIntyre's definition of a practice as a ‘coherent and complex form of socially established cooperative human activity.”’

The question remains, however, concerning the end or purpose for which business functions are integrated. In other words, in what sense can we view the integrating activity characterizing business as directed toward internal goods, rather than solely toward the attainment of profits? Moore (2002, p. 230) says in “What it means to be an excellent builder will clearly differ in some respects from what it means to be an excellent retailer, although there will be common features such as an emphasis on quality and high levels of customer service.” We maintain that Moore has an important insight here concerning the nature of business, as an activity that is focused primarily on benefiting or creating value for customers, rather than primarily achieving a notion of excellence that has no reference to customer value. Building upon Moore's insight, we argue below that excellence in business is a matter of integrating resources of various sorts in order to achieve mutually beneficial outcomes. First, however, it should be noted that Moore fails to develop this insight further—the idea that excellence in business is about benefiting customers—and this is the point that draws criticisms from Beadle (2008).

Beadle (2008, p. 232) criticism of Moore focuses on a failure to expand on the nature of the internal goods specific to business:

Moore gives no example of the excellences of business qua business save that of “customer service.” The other internal goods he highlights: “quality” [...] and “the exercise of practical skills, the stimulation that the competitive situation affords, pride in accomplishment and the personal dignity that derives from a job well done” [...] are generic descriptors equally applicable to other practices.

Although we agree that more needs to be said about the nature of the internal goods specific to modern business, we think that Beadle's criticism is not decisive for two reasons. First, we agree that quality and customer service are both too vague and too specific. We claim instead that one key internal good of business *qua* business is mutually beneficial exchange attained through the creation of customer value. This may be achieved through high-quality or innovative products, or products that are produced more efficiently and, thus, available to customers who would otherwise be deprived of their benefits. Likewise, customer service is a potential source of value in some cases, but not all. However, value for customers is a general requirement of excellent business, and we can say that any business that fails to benefit customer on a regular basis, at least to some degree, is a sham, a matter of rent-seeking rather than a real business.

Second, Beadle (2008, p. 232) calls many of Moore's terms for internal goods “general descriptors.” The implication of Beadle's claim is that Moore's purported internal goods are not specific to business but are rather applicable to many different practices. Consider, however, how MacIntyre (2007, p. 188) describes the internal goods of the practice of chess; these include “a certain highly particular kind of analytical skill, strategic imagination and competitive intensity.” MacIntyre explains further that the phrase “highly particular kind” is a way of pointing to the specificity of the goods in question, and that any attempt to describe the manner in which goods are specific to particular practices faces difficulties concerning the limits of language. Using this standard, we can adopt MacIntyre's phrasing and describe the internal goods of business as involving a “highly particular kind of analytical skill, strategic imagination and competitive intensity” attained while integrating resources of various sorts including different business functions, following Moore (2002, p. 24), in order to create value for customers.

There is one complication to this account of the internal goods of business conceived as a practice. Arguably it conflates business with social enterprise. Where the latter is focused primarily on the benefits it provides to customers, including especially addressing the needs of marginalized or disadvantaged persons (Santos, 2012, p. 348), business generally aims to create value for customers while earning a profit. Accordingly, within the tradition of classical liberalism going back to Smith (2003), commerce or business has been seen as focused on *mutual* benefit (Bruni and Sugden, 2013; Sugden, 2015; Otteson, 2019). That is, from this perspective, business involves bringing about mutually beneficial exchanges between willing transaction partners. Thus, we can restate our account of the internal goods of business as involving a distinct mode of analytical skill, strategic imagination, and competitive intensity focused on integrating resources of various sorts in order to facilitate mutually beneficial exchanges between transaction partners. As such, our account of business as a practice is peculiar in that we maintain that its distinct ideals can be helpfully understood from within the liberal tradition (see MacIntyre, 1988; Tomasi, 2012). We revisit this issue in the concluding discussion.

## Business and strategy

Relatively recent trends within strategic management—trends ensuing since the publication of the first edition of *After Virtue* in 1981—provide broad support for our account of business as a practice, and they conflict with the dominant account of firms as practice-institution combinations within MacIntyrean business ethics (Moore, 2005, 2017; Beadle and Moore, 2006; Moore and Beadle, 2006; Tsoukas, 2018). Though it goes without saying that research in strategy has not focused specifically on the question of whether business is a MacIntyrean practice, we argue nevertheless that many prominent perspectives in the field support our account of the internal goods of business.

The first perspective that is relevant in this regard is the knowledge-based view of the firm (KBV) (Kogut and Zander, 1992). This strand of research has emphasized the distributed and heterogeneous nature of knowledge within the firm (Tsoukas, 1996) with the key task being the integration of this knowledge (Grant, 1996). This is important both because it supports the notion that the practice of business is centered upon integrating different business functions or forms of knowledge, and because it suggests that the notion of the firm as centered upon a single core practice, as Moore (2017) and others (Beadle and Moore, 2006; Tsoukas, 2018) have theorized, often misrepresents the nature of the firm. Firms are, instead, typically focused on integrating different forms of knowledge, or different “knowledge domains” (Grant, 1996). As such, firms often contain numerous practices, involving various business functions, i.e., finance and accounting, as well professionals of various sorts, i.e., lawyers, engineers, human resource managers, etc., and, depending on the industry, possibly even practitioners from different sciences, including for example, specialists in ecology and environmental science, crafts, or professions.

This focus on firms as containing multiple practices is further supported by research centered upon collaboration and joint production (Foss and Lindenberg, 2012; Birkinshaw et al., 2014; Adler and Heckscher, 2018). This work highlights the fact that firm-level goals focused on dynamic value creation are typically different from the goals of sub-organizational level groups or practices. Again, consider the firm as a practice-institution combination as in the MacIntyrean perspective (Moore, 2017) in the light of this research. From the MacIntyrean perspective, when all is functioning properly, core practice members work together to realize shared internal goods of the practice. But very often this is not how production or innovation occurs. Instead, within joint production, members of different practices must learn to interact dynamically in order to create value or innovate, adapting their actions and goals to the changing demands of members of other practices (Adler, 2015). At Apple, for example, software engineers may have to work with design specialists, marketing specialists, and hardware experts to develop new products in a synergistic process (Kocienda, 2018). And product development at Apple is not unusual in this regard. In many cases, firm members may do their best work by collaborating with members of different practices (Adler, 2015). Thus, business should be seen as a distinct practice that transcends any of the particular productive practices it contains, dynamically shaping and integrating them to find new, better, or more efficient ways to create value for customers. At least, this is the upshot of much recent work in strategy focused on collaboration.

The focus on distinct, organization-level goals, involving some form of value creation, i.e., benefits to customers, is further emphasized in the theory-based view of strategy (Felin and Zenger, 2017). From this perspective, firms embody distinct theories of value creation, what Felin and Zenger (2016) refer to as a *theory for the firm*. This is a unique, firm-specific conception of value and a distinct notion of the way that a firm can deliver this value to customers. What is important is that theories of value creation, typically developed by an entrepreneur or entrepreneurial team will normally involve novel combinations of resources, forms of knowledge, and business functions which are then integrated in unique ways to create novel forms of value, i.e., new or improved products or services, or cheaper and more efficient versions of existing products or services. As such, firms' theories of value creation will often combine multiple practices, when, for example, an entrepreneur sees some way of meeting or anticipating customer needs through a new combination of knowledge, skills, and resources. Again, Apple provides a relevant example, since the development and production of their products typically require contributions from participants in many different practices. But Apple is not unique in this regard; consider automobile manufacturers, pharmaceutical companies, or aviation firms like Boeing or Airbus. Inevitably, design and production in these industries draws upon knowledge, skills, and resources from multiple practices—including scientists and engineers of various types—which must also be combined with contributions from members of professions like accounting, finance, and law.

However, modern firms cannot focus only on value creation, but must also focus on value capture. That is, firm decision-makers face financial constraints such that concerns regarding value capture play an important role in determining feasible strategies for pursuing value creation. For example, the decision to develop novel products or processes for producing existing products may be made, in part, because effective “isolating mechanisms” (Rumelt, 1984) can be identified—that is, mechanisms that make it harder for other firms to erode the focal firm's competitive advantage. The competitive nature and large size of many firms in modern business requires that firms pursue profitable strategies based on the principle of mutual benefit. As Liebeskind (1996, p. 104) says, “The managerial strategies of firms, then, can be understood as representing rent-seeking behavior, directed both at innovation—the discovery or creation of new processes and products—and at the discovery or creation of 'isolating mechanisms' that serve to protect a firm's innovations from expropriation or imitation by rivals…”

The link between value creation and value capture, or mutual benefit, is evident in many recent discussions of stakeholder theory within strategy, where the key idea is that stakeholders must be adequately incentivized. Stakeholders must have a sufficient right to capture portions of the value created in order to be motivated to make the specialized investments in human capital necessary to create value within a specific firm (Barney, 2018; Stoelhorst, 2021). In other words, value creation and value capture are intertwined such that individuals are unlikely to engage in the former if they are not sufficiently able to engage in the latter. The upshot, for our purposes, is that the concept of mutual benefit (Bruni and Sugden, 2013; Sugden, 2015; Otteson, 2019) better captures two aspects of modern business practice: first, the important focus in firms on value creation, especially in providing benefits to customers; second, the necessary emphasis on value capture, and the ethos of the typical firm member who is happy to produce excellent goods and services, but also wishes to be remunerated fairly for doing.

Bringing these ideas together with our previous discussion of business as a practice, we can now say that business is a practice focused on identifying and implementing novel ways of integrating various core and auxiliary practices, involving heterogeneous resources, knowledge, skills, and business functions, to facilitate value creation and value capture. This definition builds upon Moore (2002, p. 24), and it incorporates insights from the contemporary literature on strategic management. In order to provide more elaboration of this account of business, and in order to highlight differences with regard to the practice-institutions framework (Beadle and Moore, 2006; Moore, 2017), we next consider the role of the virtues in supporting modern business practices.

## The virtues of value creation

Our review of the strategy literature has emphasized the strategic importance of collaboration and innovation in the practice of business. Although several different kinds of virtue contribute to, and are supported by the activities of collaboration and innovation in firms, we choose three interrelated virtues to discuss, namely, creativity, cooperation, and sustained focus.

### Creativity

Creativity is self-evidently important for innovation and value creation, but its nature is rather mysterious, and its status as a virtue has been disputed. For example, although Linda Zagzebski considers originality and creativity to be virtues, she considers them to be inherently opposed to the processes of habituation characteristic of other virtues (Zagzebski, 1996, p. 123–125). The problem is that in order for an idea to be considered original, it must break from what would be, or seem to be, a normal or habituated response. A more helpful account of creativity, at least in the context of business, is provided by Christine Swanton's pluralistic account of virtue. For Swanton, creativity is not technically a virtue but, rather, a “‘mixed' form of moral acknowledgment … which informs a wide range of virtues … contain[ing] both externalist and internalist elements” (Swanton, 2003, p. 161–162). Explicitly referencing MacIntyre's account of the internal goods of a practice, Swanton says that creativity, on the one hand, “is a success word” involving tasks, products and outcomes that can be measured and evaluated according to criteria that include novelty, surprise and value (Swanton, 2003, p. 165). On the other hand, however, creativity also implies an underlying disposition of the person who creates an original work—a disposition that involves “intelligence, imagination, skill, inventiveness, or talent” and typically involves “excitement, spontaneity, feelings of discovery, delight, joy, and other emotions” (Swanton, 2003, p. 163).

In the context of business, the virtue of creativity is crucial because processes of value creation require that actors within firms collaborate together in a way that enables firms to develop unique capabilities that cannot be imitated by other firms. That is, for firms to be successful, they must create value for customers, and the virtue of creativity, as exhibited by firm actors in their collaboration together, contributes in a crucial way to excellence in this endeavor. Moreover, creativity should not be understood as merely an intellectual virtue, but also as a moral virtue that is related to the other virtues. This connection has two implications in business practice. First, it is important to understand creativity as being deeply intertwined with social (or collective) endeavors, because modern business activity involves the cooperation of many different actors. This means that creativity is intrinsically linked to cooperative virtue, as discussed in more detail below. In this vein, some scholars have argued that creativity in business should be understood as a collective virtue, in order to avoid the mistake of promoting “a perverse obsession with individual visionaries and creative geniuses” (Astola et al., 2022).

The second implication that follows from creativity being related to the other virtues is important for differentiating between genuine business activity and mere simulacra. That is, for business to be a practice, it is necessary to adopt at least modest version of the unity-of-virtues thesis, in order to rule out business activity that does not contribute—or cannot be expected to contributed—to the flourishing of its customers. This condition rules out business activity that is deceptive or harmful, either to customers or to society. Unvirtuous forms of business activity fail to create genuine customer value and, therefore, fail to comport to the standards of excellence in business practice. Here, however, it is important to note, again, that our conception of mutual benefit is liberal rather than neo-Aristotelian, and this means we do not consider it the responsibility of business practitioners to impose their own substantive views regarding the Good onto their customers. We revisit this liberal aspect of our theory in our concluding discussion.

### Cooperation

The virtue of cooperation in business is helpfully framed within a context of collaborative problem solving. Following Swanton (2003, p. 254–258), we refer to this activity as “constraint integration.” On Swanton's account, practical reasoning characteristically involves actions where various normative constraints apply, often pointing in opposite directions (p. 273–291). In this context, a key role of virtue is to determine how to coherently integrate these various constraints. In the context of individual decision-making, the problem of constraint integration is handled primarily by the virtue of practical wisdom. In the context of social dilemmas, constraint integration not only requires practical wisdom but also various kinds of cooperative virtue. For example, dialogical virtue is needed to facilitate information sharing and deliberation so that, for example, viewpoints and ideas will be expressed non-dogmatically in order to encourage the sharing of diverse sources of information and perspective (Swanton, 2003, p. 264–266). Constraint integration is often achieved by reconceptualizing constraints. That is, rather than interpreting constraints in a rigid way, collective deliberation makes it possible to reconceptualize the purpose or aim of various constraints, and thus make it possible to see how the underlying aim of certain constraints or combinations of constraints could be accomplished by alternative means.

A key role of cooperative virtue in business is to contribute to innovation, which is accomplished, as Stacey and Eckert (2010, p. 241) put it, not by out-of-the-box thinking, but by “being in the right box … designed by the designer's active construction of a problem to solve.” Or, as argued in a similar vein by Lombardo and Kvålshaugen (2014, p. 2), constraint handling in organizational contexts should be conceived so that constraints are understood as being “inherent in creative action,” rather than “external factors … excluded from the conceptualization of the creative act itself.” Especially in contexts of innovation and entrepreneurship, value creation occurs when disparate perspectives, resources, and expertise are involved. This implies that the practice of business is characteristically a form of activity that functions across domains. Accordingly, our account of business can be contrasted with Beabout's (2012) conception of management as a domain-relative practice. That is, whereas Beabout argues that management comprises a MacIntyrean practice only within the bounds of a particular business domain, it is fundamental to our conception of business that its internal goods are realized by integrating knowledge and resources *across* various domains of core practices, business functions, or sources of knowledge and expertise.

### Sustained focus

A final type of virtue associated with the internal goods of business is what we term *sustained focus*, which represents a combination of the virtues of perseverance and focus. Swanton (2003, p. 260–261) defines the “virtues of focus” as involving a “disposition and ability to establish and maintain a shared focus” on a particular problem, which requires “not just acumen, discipline, sensitivity, and wisdom, but also … courage and persistence” (Swanton, 2003, p. 260). In the task of value creation, as realized through innovation and collaboration, sustained focus is closely related to a firm's strategy and raison d'être which, as discussed above in the context of the knowledge-based view of the firm (Kogut and Zander, 1992), is create value for customers through cumulative, ongoing processes of knowledge sharing and transfer.

In a similar vein, according to the theory-based view of strategy (Felin and Zenger, 2016: p. 260–261), firms develop distinct value-creating capabilities by attending to a distinctive “theory,” comprised of a “directed and focused” way of mapping the world, involving questions, problems, and hypotheses. Through sustained focus and experimentation, a firm's strategy-as-theory can reveal “heretofore unattended features or characteristics of reality” and “new possible uses and functions” of existing resources (Felin and Zenger, 2016, p. 260–261). In order for a theory to create value for customers, firm actors must continually update and test a unique set of hypotheses in a sustained and generative way. In the case of Uber, for example, initial conjectures regarding the possibility of a novel ride-sharing theory were formulated. These initial conjectures led to further questions, inquiry, and theory-refinement regarding problems, including: “riding with strangers, the facilitation of skillful navigation for less experienced drivers, managing efficient payment, and effective driver onboarding” (Felin and Zenger, 2016, p. 264). Uber's success was achieved by maintaining a cooperative and sustained focus on addressing these problems, analogous to the way that excellence in scientific inquiry occurs, namely, by making conjectures, testing hypotheses, and refining theoretical models.

## Financing innovation

In order to provide further elaboration of our account of business as a practice, and the role of the virtues, we consider how finance contributes to business. The most significant contribution of finance to business is the cultivation of innovation through the provision of capital and integration of knowledge across different actors and domains. We build on the MacIntyrean account of finance as a practice as developed in Rocchi et al. (2021), where the aim of finance is understood in terms of bridging investors and entrepreneurs (broadly understood) in support of projects that generate goods otherwise be unattainable. We add to this account an emphasis on the informational role that finance plays, specifically in facilitating innovation through the integration of information across different business domains and market participants. Financial markets and large financial institutions are uniquely able to integrate information across different domains, thanks to the economies of scale and scope they enjoy, and the ability to raise capital and distribute risk across a large number of well-diversified investors (Bruin, 2015, p. 33). Financial institutions are, thus, de facto clearing-houses for large amounts of heterogeneous and economically valuable information (Hayek, 1945), and financial practitioners play an important role in integrating information across these different domains of business activity.

The integration of information is facilitated by the sub-practices of financial analysis and financial reporting which help managers and investors make prudent decisions regarding how much capital to allocate to various projects and firms, respectively. The prominence of passive investment strategies in the modern economy can obscure the vital and active role that financial analysis plays to the functioning of finance. However, the relative efficiency of financial markets is only maintained through the work of evaluating and integrating financial information across heterogeneous firms, where each firm represents a unique combination of knowledge, skills, and resources. Financial analysis and financial reporting facilitate the integration of information between businesses and investors by overcoming the asymmetric information problem between those inside and outside the firm. Entrepreneurs, managers, and members of core practices work within the firm, and possess valuable tacit (Tsoukas, 1996) or “soft” knowledge (Edmans et al., 2016) regarding value creation possibilities, whereas investors who possess capital resources are located outside the firm, and have only limited information regarding the uses and potential uses of their capital. The role that financial practitioners play in overcoming this problem can be seen more clearly by considering two specific examples of financial practice. These examples are chosen to highlight the contribution of finance to business by promoting innovation.

### Venture capital

Venture capital (VC) investors play a particularly important role in fostering innovation, with more than 60% of total R&D spending and 90% of patent value in US publicly traded firms coming from firms that originally received backing from VC investors (Gornall and Strebulaev, 2021). Whereas specific actions of VC investors remain fairly opaque to scholars (Hawk, 2018), results of new survey data provide important insights regarding ways VC investors collaborate with entrepreneurial management teams. In evaluating early-stage ventures, the most important factor that VC investors consider is “the quality and the experience of the management team,” mentioned by 95% of respondents (Gompers et al., 2020). In a similar vein, VC investors cite the importance of evaluating the level of trust that exists between team members (Bottazzi et al., 2016). Evaluating trust is not based on hard or tangible forms of knowledge; rather, VC investors rely on soft or tacit forms of knowledge obtained through direct dialogue with entrepreneurs and fellow VC investors, and through their own personal experiences and expertise in making practical judgments regarding prospective ventures. After analyzing financial metrics and forecasts, 44% of VC investors admit that they ultimately make “gut investment decisions” (Gompers et al., 2020). These findings suggest that the virtues of practical wisdom, creativity, and cooperation play an important role in the value-generating activity of information integration that occurs in the collaboration between VC investors and entrepreneurs.

In addition to applying their expertise to identify and evaluate new ventures, VC investors also provide valuable advice to entrepreneurial teams after they receive initial financing. This direct form of cooperative engagement is important for ensuring that investor-entrepreneur collaboration will be successful in integrating information across their respective business functions and expertise, in order to realize “post-investment value-add” (Gompers et al., 2020). Also, to maximize the chance of successful implementation, VC investors must determine the appropriate amount, timing, and contractual stipulations for each round of VC financing, including details pertaining to compensation, risk-sharing, control rights, and equity stakes. In applying their particular knowledge, expertise, and skill in making these judgments, VC investors merge their own unique insights with the specific knowledge and skills from the entrepreneurial teams with whom they work. Collaboration with VC investors also helps entrepreneurs obtain capital and knowledge-based resources necessary for successful implementation of their innovative ideas through the prudent allocation of risk, capital, and incentives. This sustained focus on collaborative entrepreneurial ventures makes it possible for VC investors to identify the entrepreneurial talent and novel resource combinations that have the most value-generating potential.

### Dedicated investors

A second group of financial practitioners who facilitate innovation by integrating information are dedicated investors. Dedicated investors are active institutional investors who take large, long-term positions in a small number of firms. Dedicated investors expend more effort than other investors on a focused group of firms in order to gain a deeper understanding of firm resources and policies (Bushee, 1998; Eccles et al., 2014; Shi et al., 2017). Dedicated investors also engage in direct forms of dialogue and collaboration with firm decision makers regarding firm-level practices and policies (Oehmichen et al., 2021). Through these activities, dedicated investors are afforded unique insights regarding firm-specific resource combinations, which are particularly helpful for evaluating the innovative potential of multiple core practices in large and complex firms and industries (Oehmichen et al., 2021).

Engagement with firm decision makers can occur in a variety of ways, such as working with proxy advisors, who “collect information, perform delegated monitoring, and use their expertise and experience to make informed voting recommendations” (McCahery et al., 2016, p. 2926). Importantly, these recommendations are not blindly accepted, but are used by dedicated investors in forming unique judgments regarding policies they vote on Ertimur et al. (2013). By engaging with firm decision makers, dedicated investors are thus able to integrate knowledge drawn from their own particular background, history, and experience with the diffuse knowledge collected by proxy advisors, and with the various forms of knowledge possessed by other firm decision makers, including decision makers internal to the firm more directly engaged in the firm's core practices.

In addition to facilitating the integration of information between investors external to the firm managers, directors, and members of core practices inside the firm, dedicated investors are also well-positioned to help firms make long-term commitments. In doing so, dedicated investors help to insulate sub-organizational goals and standards of excellence of core practices from short-term pressure exerted by financial markets. For example, dedicated investors can facilitate long-term commitments by collaborating with firm decision makers to shape governance policies that feature a high degree of “failure tolerance” (Manso, 2011; Chen et al., 2016), which reduces myopic career concerns of managers (Aghion et al., 2013). Implementation of failure tolerance policies have been shown to be more effectively when managers or directors have a high level of expertise in core or auxiliary practices, which facilitates knowledge sharing across domains (Brav et al., 2018). One way that long-term commitments promote value generation is by encouraging managers to be bolder and more creative in implementing firm-level strategies, resulting in strategies that have been found to be more distinctive (Oehmichen et al., 2021), more innovative (Chen et al., 2016; Brav et al., 2018), and more committed to environmental and social sustainability (Eccles et al., 2014). To achieve these results, dedicated investors, firm managers, and directors must jointly exercise the virtues: creativity, to support innovation; cooperation, to support information sharing; sustained focus, to support long-term value creation.

Rocchi et al. (2021) argue that finance is a domain-specific practice. However, their account misses the important role that finance plays in integrating information across different domains. Innovation, and thus the creation of novel internal goods that benefit customers, requires the integration of information and the production of knowledge across heterogeneous domains. This type of integration is facilitated by collaboration between investors, entrepreneurs, and managers. Financial practitioners make an important contribution to this process by providing capital and integrating information from disparate sources that spurs innovation and value creation that would otherwise not occur.

## Concluding discussion

Our arguments concerning the nature of business and our review of current discussions of business in strategy and finance highlight difficulties for the MacIntyrean account of firms as practice-institution combinations, as defended by Moore (2017) in much of his later work. These difficulties are threefold. First, the account of firms as centered around a core practice does not cohere very well with prominent discussion of the firm and its key attributes and functions within contemporary management research. From our review of this literature, albeit cursory, it is clear that business typically involves integrating various modes of knowledge, or practices, in order to create value, rather than focusing on a single core practice. Second, it is clear that value capture shapes the way that firms approach value creation. As such, it is not plausible to suggest that firms subordinate external goods to internal goods. Instead, it is much more plausible to view firms as focused on mutual benefit through value creation and value capture.

This leads to a third difficulty: the neo-Aristotelian perspective that informs the work drawing upon the MacIntyrean practice-institution framework leaves little room to say anything particularly positive about the role of profits in business. From this perspective, profits are seen as a crucial means to create internal goods but they are not viewed as valuable in themselves, something managers or entrepreneurs have reason to pursue on their own. However, it seems likely that the average business person, not to say the average management scholar, takes a different view, treating profits or financial gain as worth pursuing because money is a useful means to many different ends—not merely the internal good of the practice, but any of the ends that make up a meaningful life. Thus, financial success, we claim, is worth pursuing because it enhances a person's autonomy (Wyma, 2015). This account of financial success as choice-worthy because it contributes to autonomy does not negate our core thesis, that there are internal goods in the practice of business; however, it coheres more easily with a liberal rather than neo-Aristotelian framework. The same is true regarding our focus on mutual benefit. Thus, the account developed here may be seen both as a classical liberal (Otteson, 2019) appropriation of the MacIntyrean framework and a challenge to the neo-Aristotelian, MacIntyrean approach to offer a more plausible account of the nature of financial success and its role in a virtuous life. Interestingly, Santori (2021) has recently argued that Aquinas gives mutual benefit a central role in his account of commerce, recognizing reasonable financial gain as a legitimate goal. From this perspective, it may be possible to develop a Thomist account of business that both gives greater legitimacy to the pursuit of external goods. Such an account would necessarily treat business as sui generis, a practice that aims to facilitate mutually beneficial exchange. We argue that any such account would do well to return to Moore's (2002) early account of business as a practice, enriching with later work in MacIntyrean business ethics, as well as through engaging with organization research.

Further, we have highlighted the role of the virtues of creativity, cooperation, and sustained focus in ensuring that collaboration between various business actors results in value creation, and not just value capture. Virtue in business is also crucial for ensuring that business activity remains centered on mutual benefit (Bruni and Sugden, 2013), rather than the exploitation of customers through various forms of asymmetric information, or the exploitation of other stakeholders through negative externalities. Although much more remains to be said on this topic, we hope to have shown that Moore's (2002) early account of business as a practice offers important insights and promise for appreciating business on its own terms, and for recognizing its inherent moral texture. Stated more broadly—using Hegelian terminology—we aim to show, at least in outline, that business is a form of *ethical life*, a thick normative context within which individuals are embedded such that their aspirations, motivations, and moral judgments can only be fully appreciated in its light (see Perreau-Saussine, 2022). As such, we maintain that Moore's (2002) early account of business as a practice, informed appropriately with later insights, has much to offer future research in business ethics and organization studies.

## Data availability statement

The original contributions presented in the study are included in the article/supplementary material, further inquiries can be directed to the corresponding author.

## Author contributions

All authors listed have made a substantial, direct, and intellectual contribution to the work and approved it for publication.

## References

[B1] Adler P. S. (2015). Community and innovation: from Tönnies to Marx. Organ. Stud. 36, 445–471. 10.1177/0170840614561566

[B2] Adler P. S. Heckscher C. (2018). Collaboration as an Organization Design for Shared Purpose. Toward Permeable Boundaries of Organizations? Research in the Sociology of Organizations. Bingley: Emerald Publishing Limited, 81–111. 10.1108/S0733-558X20180000057004

[B3] Aghion P. Van Reenen J. Zingales L. (2013). Innovation and institutional ownership. Am. Econ. Rev. 103, 277–304. 10.1257/aer.103.1.277

[B4] Astola M. Bombaerts G. Spahn A. Royakkers L. (2022). Can creativity be a collective virtue? Insights for the ethics of innovation. J. Bus. Ethics 179, 907–918. 10.1007/s10551-021-04833-0

[B5] Barney J. B. (2018). Why resource-based theory's model of profit appropriation must incorporate a stakeholder perspective. Strateg. Manage. J. 39, 3305–3325. 10.1002/smj.2949

[B6] Beabout G. R. (2012). Management as a domain-relative practice that requires and develops practical wisdom. Bus. Ethics Q. 22, 405–432. 10.5840/beq201222214

[B7] Beadle R. (2008). Why business cannot be a practice. Anal. Kritik 30, 229–241. 10.1515/auk-2008-0114

[B8] Beadle R. Moore G. (2006). MacIntyre on virtue and organization. Organ. Stud. 27, 323–340. 10.1177/0170840606062425

[B9] Bernacchio C. Foss N. J. Lindenberg S. (2022). The virtues of joint production: ethical foundations for collaborative organization. Acad. Manage. Rev. 10.5465/amr.2019.0389. [Epub ahead of print].

[B10] Birkinshaw J. Foss N. J. Lindenberg S. (2014). Combining purpose with profits. MIT Sloan Manag. Rev. 55, 49.

[B11] Bottazzi L. Da Rin M. Hellmann T. (2016). The importance of trust for investment: evidence from venture capital. Rev. Financ. Stud. 29, 2283–2318. 10.1093/rfs/hhw023

[B12] Brav A. Jiang W. Ma S. Tian X. (2018). How does hedge fund activism reshape corporate innovation? J. Financ. Econ. 130, 237–264. 10.1016/j.jfineco.2018.06.012

[B13] Bruin D. (2015). Ethics and the Global Financial Crisis. Cambridge: Cambridge University Press.

[B14] Bruni L. Sugden R. (2013). Reclaiming virtue ethics for economics. J. Econ. Perspect. 27, 141–163. 10.1257/jep.27.4.14111657406

[B15] Bushee B. J. (1998). The influence of institutional investors on myopic RandD investment behavior. Account. Rev. 73, 305–333.

[B16] Chen J. Leung W. S. Evans K. P. (2016). Are employee-friendly workplaces conducive to innovation? J. Corp. Fin. 40, 61–79. 10.1016/j.jcorpfin.2016.07.011

[B17] Eccles R. G. Ioannou I. Serafeim G. (2014). The impact of corporate sustainability on organizational processes and performance. Manage. Sci. 60, 2835–2857. 10.1287/mnsc.2014.1984

[B18] Edmans A. Heinle M. S. Huang C. (2016). The real costs of financial efficiency when some information is soft. Rev. Finance 20, 2151–2182. 10.1093/rof/rfw03035811416

[B19] Ertimur Y. Ferri F. Oesch D. (2013). Shareholder votes and proxy advisors: evidence from say on pay. J. Account. Res. 51, 951–996. 10.1111/1475-679X.12024

[B20] Felin T. Zenger T. R. (2016). CROSSROADS-strategy, problems, and a theory for the firm. Organ. Sci. 27, 222–231. 10.1287/orsc.2015.1022

[B21] Felin T. Zenger T. R. (2017). The theory-based view: economic actors as theorists. Strategy Sci. 2, 258–271. 10.1287/stsc.2017.0048

[B22] Foss N. J. Klein P. G. (2012). Organizing Entrepreneurial Judgment: A New Approach to the Firm, 1st Edn. Cambridge: Cambridge University Press.

[B23] Foss N. J. Lindenberg S. (2012). Teams, team motivation, and the theory of the firm. Manag. Decis. Econ. 33, 369–383. 10.1002/mde.2559

[B24] Gompers P. A. Gornall W. Kaplan S. N. Strebulaev I. A. (2020). How do venture capitalists make decisions? J. Financ. Econ. 135, 169–190. 10.1016/j.jfineco.2019.06.011

[B25] Gornall W. Strebulaev I. A. (2021). The Economic Impact of Venture Capital: Evidence from Public Companies. Available online at: https://papers.ssrn.com/abstract=2681841 (accessed June 1, 2021)

[B26] Grant R. (1996). Toward a knowledge-based theory of the firm. Strateg. Manage. J. 17, 109–122. 10.1002/smj.4250171110

[B27] Hawk S. (2018). Inside the secret world of venture capital. Insights by Stanford Business. Available online at: https://www.gsb.stanford.edu/insights/inside-secret-world-venture-capital (accessed June 20, 2022).

[B28] Hayek F. A. (1945). The use of knowledge in society. Am. Econ. Rev. 35, 519–530.

[B29] Kocienda K. (2018). Creative Selection: Inside Apple's Design Process During the Golden Age of Steve Jobs Illustrated Edition. New York: St. Martin's Press.

[B30] Kogut B. Zander U. (1992). Knowledge of the firm, combinative capabilities, and the replication of technology. Organ. Sci. 3, 383–397. 10.1287/orsc.3.3.383

[B31] Liebeskind J. P. (1996). Knowledge, strategy, and the theory of the firm. Strateg. Manage. J. 17, 93–107. 10.1002/smj.4250171109

[B32] Lombardo S. Kvålshaugen R. (2014). Constraint-shattering practices and creative action in organizations. Organ. Stud. 35, 587–611. 10.1177/0170840613517597

[B33] MacIntyre A. (1988). Whose Justice? Which Rationality? 1st Edn. Notre Dame: University of Notre Dame Press.

[B34] MacIntyre A. C. (2007). After Virtue: A Study in Moral Theory. Notre Dame: University of Notre Dame Press.

[B35] MacIntyre A. C. (2015). “The irrelevance of ethics,” in Virtue and Economy, eds A. Bielskis and K. Knight (Farnham: Ashgate), 7–21.

[B36] Manso G. (2011). Motivating innovation. J. Finance 66, 1823–1860. 10.1111/j.1540-6261.2011.01688.x

[B37] McCahery J. A. Sautner Z. Starks L. T. (2016). Behind the scenes: the corporate governance preferences of institutional investors. J. Finance 71, 2905–2932. 10.1111/jofi.12393

[B38] McCloskey D. N. (2007). The Bourgeois Virtues: Ethics for an Age of Commerce, 1st Edn. Chicago, IL: University of Chicago Press.

[B39] Moore G. (2002). On the implications of the practice-institution distinction: Macintyre and the application of modern virtue ethics to business. Bus. Ethics Q. 12, 19–32. 10.2307/3857646

[B40] Moore G. (2005). Humanizing business: a modern virtue ethics approach. Bus. Ethics Q. 15, 237–255. 10.5840/beq200515212

[B41] Moore G. (2017). Virtue at Work: Ethics for Individuals, Managers, and Organizations. Oxford: Oxford University Press.

[B42] Moore G. Beadle R. (2006). In search of organizational virtue in business: agents, goods, practices, institutions and environments. Organ. Stud. 27, 369–389. 10.1177/0170840606062427

[B43] Oehmichen J. Firk S. Wolff M. Maybuechen F. (2021). Standing out from the crowd: Dedicated institutional investors and strategy uniqueness. Strateg. Manage. J. 42, 1083–1108. 10.1002/smj.3269

[B44] Otteson J. R. (2019). Honorable Business: A Framework for Business in a Just and Humane Society. New York, NY: Oxford University Press.

[B45] Perreau-Saussine É. (2022). Alasdair MacIntyre: An Intellectual Biography. Notre Dame, IN: University of Notre Dame Press.

[B46] Rocchi M. Ferrero I. Beadle R. (2021). Can finance be a virtuous practice? A MacIntyrean account. Bus. Ethics Q. 31, 75–105. 10.1017/beq.2020.5

[B47] Rumelt R. P. (1984) “Theory, strategy and entrepreneurship,” in Competitive Strategic Management. Englewood Cliffs, NJ: Prentice-Hall), 556–570.

[B48] Santori P. (2021). Thomas Aquinas and the Civil Economy Tradition: The Mediterranean Spirit of Capitalism. New York, NY: Geoff MooreDurham University.

[B49] Santos F. M. (2012). A positive theory of social entrepreneurship. J. Bus Ethics. 111, 335–351. 10.1007/s10551-012-1413-4

[B50] Shi W. Connelly B. L. Hoskisson R. E. (2017). External corporate governance and financial fraud: cognitive evaluation theory insights on agency theory prescriptions. Strateg. Manage. J. 38, 1268–1286. 10.1002/smj.2560

[B51] Smith A. (2003). The Wealth of Nations Bantam. Cannan E, ed. (Bantam Classic: New York, NY).

[B52] Stacey M. Eckert C. (2010). Reshaping the box: creative designing as constraint management. Int. J. Prod. Dev. 11, 241–255. 10.1504/IJPD.2010.033960

[B53] Stoelhorst J. W. (2021). Value, rent, and profit: a stakeholder resource-based theory. Strateg. Manage. J. Available online at: https://onlinelibrary.wiley.com/doi/full/10.1002/smj.3280

[B54] Sugden R. (2015). Team reasoning and intentional cooperation for mutual benefit. J. Social Ontol. 1, 143–166. 10.1515/jso-2014-0006

[B55] Swanton C. (2003). Virtue Ethics: A Pluralistic View. Oxford: Oxford University Press. 10.1093/0199253889.001.0001

[B56] Teece D. J. (2007). Explicating dynamic capabilities: the nature and microfoundations of (sustainable) enterprise performance. Strateg. Manage. J. 28, 1319–1350. 10.1002/smj.640

[B57] Tomasi J. (2012). Free Market Fairness, 1st Edn. Princeton: Princeton University Press.

[B58] Tsoukas H. (1996). The firm as a distributed knowledge system: a constructionist approach. Strateg. Manage. J. 17, 11–25. 10.1002/smj.4250171104

[B59] Tsoukas H. (2018). Strategy and virtue: developing strategy-as-practice through virtue ethics. Strateg. Organ. 16, 323–351. 10.1177/1476127017733142

[B60] Wicks A. C. (1996). Reflections on the practical relevance of feminist thought to business. Bus. Ethics Q. 6, 523–531. 10.2307/3857503

[B61] Wyma K. D. (2015). “The case for investment advising as a virtue-based practice. J. Bus. Ethics 127, 231–249.

[B62] Zagzebski L. T. (1996). Virtues of the Mind: An Inquiry into the Nature of Virtue and the Ethical Foundations of Knowledge. Cambridge: Cambridge University Press.

